# Assessment of sanitary condition of services as implication for intestinal parasitic infections among prison inmates: institutional based cross-sectional study in eastern Tigrai zonal prison, northern Ethiopia, 2018

**DOI:** 10.1186/s13104-019-4449-z

**Published:** 2019-07-12

**Authors:** Fitsum Mardu, Brhane Berhe, Kebede Tesfay, Hadush Negash

**Affiliations:** 1 Unit of Medical Parasitology, Department of Medical Laboratory Sciences, College of Medicine and Health Sciences, Adigrat University, Adigrat, Ethiopia; 2 Unit of Medical Microbiology, Department of Medical Laboratory Sciences, College of Medicine and Health Sciences, Adigrat University, Adigrat, Ethiopia

**Keywords:** Adigrat prison, Assessment, Ethiopia, Intestinal parasites, Prevalence, Prison inmates, Sanitary condition

## Abstract

**Objective:**

The study aimed to assess the sanitary condition of services and its implication for intestinal parasitic infections among prison inmates in eastern Tigrai, northern Ethiopia.

**Results:**

We have assessed the availability and sanitary condition of services at Adigrat prison. Frequent water cuts and unavailability of soap in the prison have challenged prisoners and food handlers to maintain their hygiene. The living rooms were overcrowded and poorly ventilated besides to unsatisfactory kitchen rooms. The prevalence of intestinal parasites among the participants was 40% (108/270). The dominant parasite was *Entamoeba histolytica/dispar* (60, 22.2%) followed by *Giardia lamblia,* 39 (14.4%). The mixed infections of *Entamoeba histolytica/dispar* and *Giardia lamblia* were detected among 17 (6.3%) of the participants. In multivariate analysis, participants who were feeding in groups were more likely to harbor intestinal parasites than those who were feeding alone (AOR: 2.1; CI 1.05–4.3). Intestinal parasites are significant health problems to the prisoners of Adigrat prison with poor sanitation of services. Therefore, provision of necessary facilities such as hand washing basins, soaps, disinfectants, disinfestations, and food utensils could significantly reduce the burden of intestinal parasites in the prison.

## Introduction

Globally, more than 10.2 million people were detained in prisons in 2013 (144 per 100,000 populations). Overcrowding has become a global challenge to accomplish the minimum standards of services to prisoners [1]. Unless detention homes like prisons are kept hygienic, the transmission of communicable diseases is possible between the prisoners [2]. Prison centers with good accommodations like ventilated rooms and hygienic conditions have positive impact on the health of prisoners [3].

In Ethiopia, there were 111,050 (128/100,000 population) officially registered prisoners in 2015 [4]. Housing and sanitary conditions are poorly available for prisoners in the country characterized by overcrowding, poor sanitary condition, inaccessibility of sanitary materials, poor medical services, and inadequate food [3].

Nearly 88% of deaths from diarrheal diseases in developing countries are due to lack of access to sanitation, unsafe drinking water, and poor hygiene practices. Improved sanitation alone could reduce these diseases by one-third [5]. Similarly, about 60% of the disease burden in Ethiopia is related to poor hygiene and sanitation conditions [6].

Sustainable water, sanitation, and hygiene (WASH) services are critical for the prevention, control, and even elimination of parasitic diseases [7]. This is especially so in prisons where there is overcrowding and prisoners have no control of their environment [8]. Thus, it is crucial to ensure the availability as well as sanitary condition of services in prisons that serve large number of clients on a regular basis. Therefore, this study aimed to assess the sanitary condition of services and prevalence of intestinal parasites at Adigrat prison, Northern Ethiopia.

## Main text

### Materials and methods

#### Study design and setting

We conducted a cross-sectional study among prison inmates in eastern zone of Tigrai, northern Ethiopia from September to December 2018. This zone has a total population of 862,348 (2012). It is located 900 km North of Addis Ababa at a longitude and latitude of 14° 16′ N 39° 27′ E, with elevation of 2457 m above sea level [9]. There were 2300 prisoners during the study period in eastern Tigrai zonal prisons.

#### Eligibility criteria

Prisoners who served 1 month or longer were eligible to participate. We excluded prisoners who took anti-parasitic treatments within 1 month of study time and those who were unconscious.

#### Sample size and sampling technique

We used single population proportion formula $\left( {{\text{n}} = {\text{Z}}^{2}_{{\upalpha/2}}* {\text{p}}\;\left( {1 - {\text{p}}} \right)/{\text{d}}^{2} } \right)$ to calculate the sample size: n = (1.96)^2^ * 0.73(0.27)/(0.05)^2^ = 307, where ‘**p**’ is population proportion (0.73): prevalence of intestinal parasites (72.9%) among inmates of Shewa-robit prison, Ethiopia [10]. Since the total population size was less than 10,000, we applied finite population correction formula:

$${\text{n}} = \frac{\text{nN}}{{{\text{n}} + {\text{N}}}} = \frac{307 \times 2300}{2607} = 270.$$

We recruited participants by systematic random sampling technique. Considering the list of prisoners as a sampling frame, we calculated the sampling interval (9) by dividing the number of prisoners (2300) by the estimated sample size (270). Then, starting with a random number from one to nine (six in our case), we selected every sixth prisoner from the list up to the desired sample size.

#### Data collection and quality control

Data pertaining to participants and sanitation of the prison were gathered using semi-structured questionnaire and checklist, respectively. The checklist contains Yes/No questions regarding the availability and/or sanitation of the five prison services: health care service, water supply, sanitation of the compound and latrines, living cells, and kitchen. The investigators and data collectors have conducted the assessment by observation and interviewing the representatives of the prison clinic. We have trained the data collectors and oriented the participants on data and sample collection.

#### Specimen processing

Stool specimens were collected with leak-proof dry containers. Senior laboratory technologists examined the specimens by direct wet mount and following formol-ether concentration technique. They used Lugol’s iodine solution to identify cysts of intestinal protozoa.

### Statistical analysis

Narrative analysis approach was used to analyze the qualitative data about the availability and sanitation of the prison services (gathered by observation and interview). For socio-demographic and clinical data, we calculated frequencies and percentages of variables. The proportion of intestinal parasites within each category of independent variables was determined using crosstabs. Then using bivariate analyses, we tested the association of each independent variable with the outcome variable. Finally, to consider possible confounders, the independent variables were tested by multivariate regression model. For analyses, we used SPSS version 21 software. P-value less than 0.05 was considered significant at 95% confidence interval.

### Results

#### Availability and sanitation of services

##### Health care

The prisoners have access to medical care in the prison center with a dispensary accessible inside. The laboratory section in the clinic however was poorly equipped. The physicians refer sick prisoners that need further diagnosis and treatment to Adigrat General Hospital, Tigrai, Ethiopia. The laboratory technicians and nurses in the clinic give regular health education to the prisoners. Nevertheless, diarrhea has still been a health problem in the area. Besides, no mass treatment against intestinal parasites has been given to the prisoners in the last 2 years. We also observed unavailability of certain anti-helminthic medications (such as Praziquantel and Niclosamide) in the dispensary.

##### Water supply

The water source of Adigrat prison comes from the water supply of Adigrat town, Tigrai, Ethiopia. The existing water storage tank was less clean, and had insufficient pressure. The water source has not been discharging water consistently during our assessment. The showers built at each block did not also release water during our stay. Moreover, we did not observe additional water source to the prison. Prisoners could not access boilers to treat water due to fear of burns. There was no maintenance team responsible for the water distribution in the prison.

##### Sanitation of the compound and latrines

The prisoners themselves clean and maintain the toilets. They manage solid waste by burning but dispose liquid waste at open field. The types of toilets available at the prison are simple pit latrine type. The water pumps built in each toilet were non-functional during our visit. However, we have observed water filled containers (barrel type) in some toilets. The prison administration does not provide soap to the prisoners. Surprisingly, the prisoners could not access toilet at night; they defecate in plastic containers inside the cells.

##### Space and quarters

Over fifty prisoners were living in one room that is less ventilated and less clean. We have seen leftover food in the living rooms. Although the prison administration admitted regular disinfestations, there were insects and pests in the cells. White washing and cleaning of the cells has not regularly been practiced in the prison.

##### Kitchen and meals

The distance from the kitchen to the toilet is approximately 150 m. The sanitation of the kitchen room, food utensils, and the roof was in general inadequate. We did not observe any water storage tank in the kitchen. The food handlers transport water from the compound for food preparation. Besides, the absence of hand washing basins has challenged the food handlers to wash their hands. The food handlers had worn gowns and hair caps during the assessment period; but they were less clean. On the other hand, none of them had ornaments in their fingers while preparing food.

##### Prevalence of intestinal parasites among study participants

Overall, 108 (40%) of the participants were infected with at least one parasite species. The protozoan *E. histolytica/dispar* and *G. lamblia* were the predominant parasites detected among 60 (22.2%) and 39 (14.4%) of the participants, respectively. The helminthes Taenia species and *A. lumbricoides* were detected among eight (3%) and four (1.5%) participants, respectively. Twenty-four (8.9%) of the participants harbored multiple infections. The combination of *E. histolytica/dispar* and *G. lamblia* were identified among 17 (6.3%) of the participants (Table 1).

**Table 1:** Percentages of intestinal parasites species identified among participants in Eastern zone of Tigrai, northern Ethiopia, 2018

Species of parasites	Number (n = 270)	Percent
Single infections		
*E. histolytica/dispar*	41	15.2
*G. lamblia*	15	5.5
*E. coli*	10	3.7
Taenia species	8	2.9
*A. lumbricoides*	4	1.5
*T. trichiura*	3	1.1
*E. vermicularis*	2	0.7
*S. mansoni*	1	0.3
Mixed infections		
*E. histolytica/dispar* +*G. lamblia*	17	6.3
*G. lamblia *+* E. coli*	5	1.9
*E. histolytica/dispar *+* E. coli *+* G. lamblia*	2	0.7
Total	108	40

##### Factors associated with intestinal parasitic infections

Majority of the participants (115, 42.6%) were within the age group of 25–34 years; of which 34.8% (40/115) were infected with intestinal parasites. In addition, 38.2% (89/233) of the male participants harbored at least one parasite species. In bivariate analysis, none of the independent variables was significantly associated with the presence of intestinal parasites (P > 0.05). However, factors with P-value less than 0.2 in bivariate analysis (residence before imprisonment, duration of stay in the prison, frequency of hand washing after toilet, and feeding with others) were further analyzed by multivariate regression. As a result, after adjustments, participants who were feeding with others in the same meal were more likely (P = 0.036) to harbor intestinal parasites than those who were feeding alone [AOR: 2.1; CI 1.05–4.3] (Table 2).

**Table 2:** Frequency of variables, positivity of intestinal parasites, bivariate and multivariate analysis of factors among study participants in Eastern zone of Tigrai, Northern Ethiopia, 2018

Variables	Frequency (%)	Positive for IP (%)	COR (95% CI)	AOR (95% CI)	P-value
Gender					
Male	233 (86.3)	89 (38.2)	1.7 (0.85–3.4)	–	–
Female	37 (13.7)	17 (45.9)	1	–	–
Age groups					
15–24	93 (34.4)	44 (47.3)	0.7 (0.28–0.69)	–	–
25–34	115 (42.6)	40 (34.8)	1.2 (0.48–2.82)	–	–
35–44	36 (13.3)	14 (38.9)	0.9 (0.35–2.76)	–	–
≥ 45	26 (9.6)	10 (38.5)	1		
Education level					
Illiterate	100 (37)	41 (41)	0.36 (0.07–1.7)	–	–
Primary school	109 (40.4)	45 (41.3)	0.35 (0.07–1.7)	–	–
Secondary school	51 (18.9)	20 (39.2)	0.38 (0.07–2.0)	–	–
College/university	10(3.7)	2 (20)	1		
Residence before imprisonment					
Rural	181 (67)	73 (40.3)	0.95 (0.5–1.6)	0.04 (0.13–0.71)	0.006
Urban	89 (33)	35 (39.3)	1	1	
Duration in prison (year)					
≤ 1	103 (38.1)	37 (35.9)	0.7 (0.45–1.25)	0.59 (0.33–1.03)	0.067
> 1	167 (61.9)	71 (42.5)	1	1	
Finger nail status					
Untrimmed	142 (52.6)	58 (40.8)	1.07 (0.66–1.7)	–	–
Trimmed	128 (47.4)	50 (39.1)	1		
Hand washing after toilet					
Always	240 (88.9)	101 (42.1)	1	2.26 (0.87–5.88)	0.093
Sometimes	30 (11.1)	7 (23.3)	2.3 (0.98–5.77)	1	–
Wash hands with					
Water only	210 (77.8)	85 (40.5)	0.9 (0.5–1.6)	–	–
Water and soap	60 (22.2)	23 (38.3)	1		
Feed with others					
Yes	206 (76.3)	89 (43.2)	0.55 (0.3–1.0)	*2.1 (1.05–4.3)*	0.036*
No	64 (23.7)	19 (29.7)	1	1	
Source of drinking water					
Tap water	221 (81.9)	88 (39.8)	1.04 (0.5–1.95)	–	–
Bottled water	49 (18.1)	20 (40.8)	1		

1 = referent

*COR* crude odds ratio, *AOR* adjusted odds ratio, *CI* confidence interval

* Significant association

### Discussion

The availability and sanitary condition of the assessed services in Adigrat prison was generally deprived. Non-provision of soaps to the prisoners is against the standard that each prisoner has to receive a minimum of 100 to 150 g per month [11]. Hand washing with soap and water prevents many parasitic infections acquired via fecal–oral route [12]. Availability of sufficient water supply and clean toilets are also imperative for the health and hygiene of prisoners [13]. However, at Adigrat prison, there was frequent water interruption besides to the absence of additional water source.

It is necessary to keep latrines clean; otherwise, they will become means of disease transmission. Addition of disinfectants and regular washing of latrines help to eliminate the eggs of certain intestinal parasites [11]. In the study prison, nonetheless, the sanitation of toilets was poor. The absence of sanitarians and shortage of water in the toilets make prisoners at risk of parasitic infections. The use of plastic containers for defecation in the living rooms at night times is also conducive for the transmission for parasites.

Food handlers have the responsibility to maintain their hygiene and hence prepare safe food. Otherwise, they can contaminate food sources with pathogenic agents [14]. Unfortunately, the food handlers’ personal hygiene in the study site was unsatisfactory. They had worn unclean gowns and hair caps during the inspection. Moreover, we observed unavailability of hand washing basins within the kitchen rooms. The roof and walls of the kitchen rooms were unhygienic and infested with rodents and flies that can be carriers of parasites.

The higher prevalence of intestinal parasites in Adigrat prison is in line with other studies conducted in prisons [10, 15]. The dominance of protozoan parasites in this study has supported another study conducted in Midwest Brazil [16]. This may be due to the direct transmission of intestinal protozoa from contaminated food, water or fingers. These parasites have also the capacity to multiply within humans, as opposed to helminthes [17]. In this study, Taenia species were the frequently identified helminthes in contrary with findings in other studies [18, 19] where hookworms were prevalent. The mere presence of intestinal parasites among the participants indicates poor sanitation and hygiene practices. Non-provision of adequate water and soap might have aggravated the condition. In addition, food handlers with poor personal hygiene could have considerable role in the transmission of parasites to the prisoners [20, 21].

Most of the demographic and clinical factors were not significantly associated with intestinal parasitic infections among the participants. However, in multivariate regression, participants who claimed feeding with others in the same meal were more likely to harbor intestinal parasites (P = 0.036) than those who reported as feeding alone (AOR: 2.1; CI 95%). As most of intestinal parasites are transmitted via fecal–oral route [12], there might be cross contamination of parasites between the prisoners while feeding in the same meal. The untrimmed fingernails in majority of the participants might also have played significant roles in transmitting parasites. This is mainly common in protozoan parasites, which tend to infect many people from a single food contamination [17]. Therefore, prisoners even with good personal hygiene could have been infected from others whose fingers containing infective stages of parasites [22, 23].

### Conclusion

Intestinal parasites are significant health problems in the study area. Enhancing hygiene practices among the prisoners and food handlers as well as provision of necessary facilities could significantly reduce the burden of intestinal parasites among the prisoners.

## Limitations

We did not estimate the intensity of intestinal helminthes due to shortage of reagents and materials. It was also difficult to differentiate the Entamoeba species detected because of unavailability of laboratories to do this in our set up.

## Data Availability

The datasets used and/or analyzed during the current study are available from the corresponding author on reasonable request

## Abbreviations

CSA: Central Statistics Agency
WASH: water sanitation and hygiene
